# HAF drives the switch of HIF-1α to HIF-2α by activating the NF-κB pathway, leading to malignant behavior of T24 bladder cancer cells

**DOI:** 10.3892/ijo.2013.2210

**Published:** 2013-12-06

**Authors:** ZHENFENG GUAN, CHEN DING, YIQING DU, KAI ZHANG, JIAN NING ZHU, TINGTING ZHANG, DALIN HE, SHAN XU, XINYANG WANG, JINHAI FAN

**Affiliations:** 1 Oncology Research Lab, Key Laboratory of Environment and Genes Related to Diseases, Ministry of Education;; 2 Department of Urology, The First Affiliated Hospital of Medical College of Xi’an Jiaotong University, Xi’an, P.R. China

**Keywords:** hypoxia, bladder cancer, NF-κB, hypoxia-associated factor, hypoxia induced factor, epithelial to mesenchymal transition, migration, invasion

## Abstract

Hypoxia is a characteristic feature of solid tumors, leading to malignant behavior. During this process, HIF family members (HIFs) and the NF-κB pathway are activated. In addition, the hypoxia-associated factor (HAF) is reported to participate in the regulation of HIFs. However, the precise relationship among HIFs, HAF and the NF-κB pathway in bladder cancer (BC) remains unknown. In the current investigation, T24 BC cells were exposed to hypoxia, or by plasmid transfection to overexpress HAF or RelA (P65) to demonstrate their roles. The results indicate that hypoxia leads to the elevation of HAF plus activation of the NF-κB pathway, accompanied by the switch of HIF-1α to HIF-2α, resulting in the enhanced ability of malignancy in T24 cells. In order to further demonstrate the significance of this switch, HIF-1α and HIF-2α were co-transfected into T24 cells with HIF-β, respectively. The following results indicate that the T24*^hif-2α/β^* cells show enhanced ability of malignancy, accompanied by the maintenance of stem-cell markers, but the T24*^hif-1α/β^* cells show higher expression of metabolism-related genes. Boyden assays and wound-healing assays indicate the enhanced ability of malignancy for T24*^hif-2α/β^*. Thus, we conclude that on the hypoxic microenvironment, the switching of HIF-1α to HIF-2α, which is driven by HAF through activating the NF-κB pathway, contributes to the malignancy of T24 cells, accompanied by the maintenance of stem-cell markers. This provides us an avenue for understanding the progression of bladder cancer.

## Introduction

Regional hypoxia is common feature of solid tumors, leading to the induction of the transcriptional factor hypoxia inducible factor (HIF) (1). Prolonged-term exposure to hypoxia, which mimics the tumor microenvironment, drives a perpetual epithelial to mesenchymal transition (EMT), whereas short-term exposure to hypoxia induces a reversible EMT (2). The discrepancy of cell behavior change driven by differing terms of hypoxia can be regarded as the manifestation of different activated target genes induced by different HIF family members (HIFs).

HIFs are composed of two heterodimeric proteins belonging to the basic helix-loop-helix (bHLH)/Per-ARNT-Sim (PAS) domain family of transcription factors (3,4), the former contains three subunits: HIF-1α, HIF-2α and HIF-3α (5,6), in particular, HIF-1α and HIF-2α (also known as EPAS1) are the two best studied members of the HIF-α family (7), HIF-3α is a new one (8–11). The latter is also known as the aryl hydrocarbon receptor nuclear translocator (ARNT) or simply HIF-β, including HIF-1β, HIF-2β and HIF-3β (8). Although HIF-1α and HIF-2α share several common targets such as VEGF (12,13), both isoforms may regulate distinct transcriptional targets (14). HIF-1α is responsible for the regulation of genes encoding enzymes involved in the glycolytic pathway but HIF-2α targets to the genes involved in invasion such as the matrix metalloproteinase (4,14,15) or the stem-cell related genes such as Oct-4 (8,11,16–18) in certain tumors. In normal atmosphere, HIF-1α and HIF-2α are degraded by hydroxylation of specific proline residues (Pro^402^ and Pro^564^ in human HIF-1α; Pro^405^ and Pro^531^ in human HIF-2α) (3,6,19–21) within their oxygen-dependent-degradation domain (ODD) by prolyl hydroxylases (PHDs) in the PHD-pVHL system, but in hypoxic environment, the PHDs are degraded, leading to the stabilization of HIF-1α and HIF-2α, rendering HIF-α capable of dimerizing with HIF-β binding to the hypoxia-responsive DNA element, resulting in the activation of hypoxia-responsive genes (7). Besides the above, HIF-α can be regulated by other factors, such as hypoxia-associated factor (HAF) (8) and the nuclear factor-κB (NF-κB) pathway.

HAF, also known as SART1_800_ (16), is reported to be able to bind to and induce the degradation of HIF-1α in PHD-pVHL-independent way (16). But this binding of HAF to HIF-2α leads to its elevation instead degradation (6,16). In addition, HAF is elevated in many solid tumors, such as breast cancer, and brain tumors, etc.

The NF-κB family is composed of structurally homologous transcription factors, including NF-κB1, NF-κB2, RelA, RelB and c-Rel, which bind to IκB enhancers as homo- or heterodimers (19,22,23). van Uden *et al* (23) reported that the HIFs could be upregulated by NF-κB, and they found the binding site of NF-κB in the promoter region of the HIFs. Degradation of IκB leads to the activation of the pathway, resulting in the nuclear translocation of the NF-κB complexes, predominantly RelA/P50 (P65/P50) and P50/c-Rel dimers (24). This activation occurs in inflammation as well as in the progression of cancer and hypoxia.

Herein, we report that in the bladder cancer T24 cells, prolonged exposure to hypoxia induces the elevation of HAF, resulting in the switch of HIF-1α to HIF-2α, the process of which is mediated by NF-κB pathway. This leads to more malignant behavior and maintenance the stem-cell markers of T24 cells, giving us a further clue to understand the mechanism for the progression of bladder cancer.

## Materials and methods

### Western blotting

Cells were harvested at 80% confluence, and washed with cold PBS three times. Total cellular protein lysates were prepared with RIPA buffer [50 mM Tris (pH 8.0), 150 mM NaCl, 0.1% SDS, 1% NP40 and 0.5% sodium deoxycholate] containing proteinase inhibitors [1% cocktail and 1 mM PMSF, both from Sigma (St. Louis, MO, USA)]. Nuclear protein was prepared using the kits (lot no. BSP001) obtained from Sangon Biotech Co., Ltd. (Shanghai, China) strictly according to its protocol. Total of 30 *μ*g of protein was separated by 10% SDS-PAGE and transferred to nitro-cellulose membranes. The membranes were blocked at room temperature for 1 h with 5% skim milk in Tris-buffered saline without Tween-20 (pH 7.6, TBS). Polyclonal antibodies were applied at different dilutions (Table I) by 5% skim milk in TBS at 4°C overnight, followed by washing with TBST (with Tween-20, pH 7.6). Membranes were incubated with secondary antibodies (supplied by Licor, Rockford, IL, USA) coupled to the first antibody at room temperature in the dark for 1 h, followed by washing as above in the dark, drying with neutral absorbent paper and scanning by Odyssey detection system (Licor). MG-132 (Sigma) was used to inhibit the proteasome-dependent degradation if necessary (10 *μ*M, 4 h before the protein harvest). GAPDH (for total cell fraction) and Hist1H1a (for nuclear fraction) were used as the loading controls.

### Real-time PCR

Total RNA of the related groups of the cell was isolated using TRIzol reagent (Invitrogen, Carlsbad, CA, USA) and quantitated by absorbance at 260 nm. RNA (2 *μ*g) was reverse transcribed using Revert Aid™ First Strand cDNA Synthesis kit (MBI Fermentas, St. Leon-Rot, Germany) according to the manufacturer’s protocol. For real-time PCR, we used the SYBRR Premix Ex Taq™ II system (Takara Biotechnology Co., Ltd., Dalian, China) and the Bio-Rad CFX96^TM^ Real-time system (Bio-Rad, CA, USA). SYBRR Premix Ex Taq II (12.5 *μ*l), 1 *μ*l primer (10 *μ*M, primers see the Table II), 200 ng cDNA and 9.5 *μ*l DD water mixed together, following the second stage, pre-degeneration for 95°C, 30 sec, one repeat, and PCR reaction, 95°C 5 sec followed by 60°C, 30 sec, 35 repeats, and the third stage of dissociation, 95°C, 15 sec followed by 60°C, 30 sec, and another 95°C, 15 sec, then the data were collected. GAPDH was used as the internal control.

### Cell culture

Human bladder cancer cell line T24 and J82 were obtained from ATCC (American Type Culture Collection, ATCC, USA) and cultured in the DMEM (Invitrogen) supplemented by 10% FBS (Invitrogen). For normal condition, the cell was cultured in the atmosphere with 5% CO_2_ at 37°C. For mimicking the hypoxic conditions, the cell was cultured in an atmosphere with 1% O_2_ and 99% CO_2_ at 37°C (Incubator: Thermo Scientific, Germany).

### Boyden chamber assay and wound healing assay

Migration and invasion were tested by Boyden chamber assay, the chambers were obtained from Millipore (Millipore, Switzerland). For migration assay, 0.2 ml FBS-free DMEM medium suspension with 10,000 cells was added to the upper chamber in a 24-well plate, and 0.8 ml FBS-free DMEM was added to the lower chamber. After 12 h of incubation, the chambers were washed with PBS (pH 7.4) three times to remove the cells in the upper chamber and were fixed with 4% formalin for 5 min, then stained with crystal violet (0.01% in ethanol) for 25 min followed by washing three times. The cells were counted in an inverted microscope, and five visions were randomly taken at ×200, the average number of the cells were analyzed; for the invasion assay, the suspension of the upper chamber contained 0.2 ml mixture (FBS-free DMEM/Matrigel=8/1, Matrigel, Sigma) and 10,000 cells, the incubating times was 36 h, other steps were the same as the migration test.

Wound healing assay was carried out by scratching a 6-well dishe with a 10-*μ*l pipette tip when the dish was at 80% confluence (including T24*^hif-1α/β^*, T24^hif-2α/β^ and T24*^vec/vec^*). The width of the scratches was compared at 0, 12 and 24 h after scratching.

### Construction of a stable clone cell line

In order to understand the role of HAF and NF-κB in T24 cell, the *pFRT/TO/HIS/FLAG/HA-SART1* and *gfp-rel a* (Addgene plasmid 38087 and 23255, http://www.addgene.org), two plasmids were transfected into the T24 cells, respectively. Lipofectamine™ 2000 (Life Technologies, USA) was used for transfection strictly according to its protocol, and selected by Blasticidin and G418 (8 and 600 *μ*g/ml) respectively.

For understanding the roles of HIFs, *pcDNA3.1-hif-1α* and *pcDNA3.1-hif-2α* (Addgene plasmid 18949 and 18950, http://www.addgene.org) were cotransfected with *pCMV-hif-β* (pCMV-HIF-β-hygro, HG13010-M, Sino Biological Inc. China) into the T24 cell, respectively. Both the subclones containing *hif-1α/β* and *hif-2α/β* were selected by G418 plus hygromycin (600 and 80 *μ*g/ml, respectively).

### Inhibition of NF-κB pathway

Pyrrolidine dithiocarbamate (PDTC) as an inhibitor of NF-κB pathway was obtained from Sigma (Sigma-Aldrich, USA) and the final concentration was 10 *μ*M in the medium for the last 24 h before the protein/total RNA was extracted or Boyden chamber assay. In addition to PDTC, siRNA for knocking down the expression of p65 was used in parallel experiments. siRNA was transfected into T24*^haf^* cells with Lipofectamine 2000 according to its protocol, and the scrambled sequence was used as a control.

### Immunofluorescence staining for nuclear translocation of NF-κB

After designated treatment, the cells were washed three times with cold PBS (pH 7.4) followed by fixing with 4% paraformaldehyde for 15 min, permeabilized in 0.5% Triton X-100 for 10 min, and incubated in 1% BSA blocking solution for 1 h. Fixed cells were incubated ovenight in 4°C with rabbit anti-human-P65 in 1% BSA. Cells were washed and incubated with mouse anti-rabbit TRITC (Red) IgG antibody (Santa Cruz, USA) diluted 1:100 in blocking buffer for 1 h. Nuclei were stained with DAPI for 5 min. Cells were examined with a fluorescent microscope equipped with narrow band-pass excitation filters to individually select for red, and blue fluorescence. Cells were observed through the Image Pro Plus system mounted on a fluorescent microscope (Olympus, Japan), the experiment was repeated thrice.

### Statistical analysis

ANOVA test was used for analyzing the discrepancy of three or more than three groups. The Student’s t-test was used to detect any statistically significant difference between two groups. P<0.05 was considered statistically significant.

## Results

### Hypoxia contributes to EMT and the enhanced ability of migration/invasion, accompanied by the elevation of HAF

**Figure 1.**
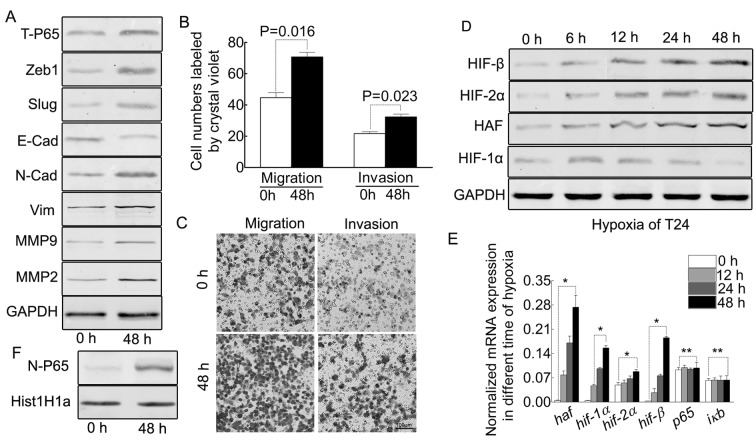
Hypoxia induces the enhanced malignancy and activation of NF-κB in T24 cells, resulting in the elevation of EMT related genes and HIFs. (A) Western blotting showing 48-h hypoxia induces the upregulation of MMP_2_, MMP_9_, N-cadherin, vimentin, Slug and Zeb1 but downregulation of E-cadherin. (B) Statistical chart of the Boyden chamber assay suggests that the ability of migration/invasion is significantly enhanced after 48-h hypoxic exposure. (C) The representative figures of Boyden chamber assay. (D) Western blotting showing the increased HIF-2α, HIF-β and HAF but decreased HIF-1α by prolonged hypoxia exposure. (E) Real-time PCR shows that the prolonged hypoxia exposure induces the elevation of *hifs* and *haf*, but has no effect on the *iκb* and *p65*. (F) Western blotting indicating the nuclear accumulation of P65 induced by hypoxia.

Tumor regional hypoxia is a common feature in solid tumors, leading to behavior change of the tumor cells in order to fit the microenvironment. Based on this, we mimicked the hypoxic environment under the condition of 1% O_2_ supplemented with 99% CO_2_ in order to observe the behavior change of our T24 cells. As indicated in Fig. 1A, the oxygen starvation for 48 h indicates EMT of T24 cells, accompanied by enhanced ability of migration/invasion (Fig. 1B and C). Previously it was reported that prolonged-term hypoxia induced the expression HAF in other tumors, our results proved this point in bladder cancer T24 cell (Fig. 1D and E).

### Prolonged hypoxia results in the elevation of HIF-2α and HIF-β but decreases HIF-1α in protein, however, increasing all the HIFs in mRNA

As reported, the prolonged-term of hypoxia led to differing expression profiles of the HIFs (11,16). Based on this, we detected all the HIFs in the different hypoxic time-points in T24 cells. As indicated by Fig. 1D, HIF-2α and HIF-β were increased from 0 to 48 h, but HIF-1α was decreased after 18 h. However, the mRNA of all the HIFs were increased (Fig. 1E).

### HAF contributes to EMT and the enhanced ability of migration/invasion in T24 cells

**Figure 2.**
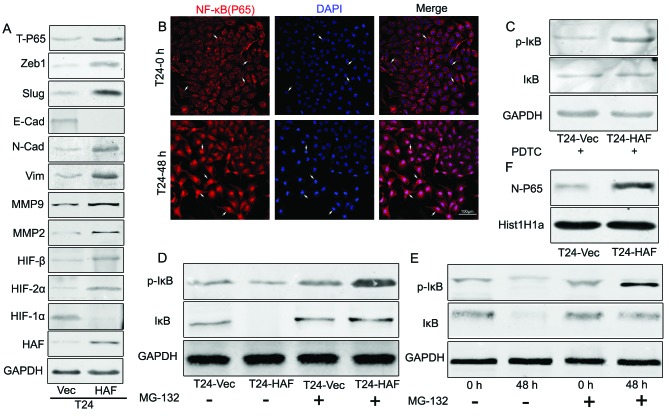
Hypoxia induces the nuclear translocation of NF-κB and the degradation of IκB/p-IκB, which also occurred in the T24*^haf^* cells, resulting in the EMT and elevation of NF-κB. (A) Western blotting showing HAF induces the upregulation of N-cadherin, vimentin, Slug, Zeb1, MMP_2_, MMP_9_ and T-P65, but downregulation of E-cadherin and HIF-1α, accompanied by the elevation of HIF-2α, HIF-β. (B) Immunofluorescence of the nuclear translocation of P65 induced by prolonged hypoxia. (C) Western blotting indicating the elevated p-IκB in T24-HAF cells in the presence of PDTC. (D) Western blotting showing the HAF-overexpression contributing to the elevation of p-IκB but without visible change of IκB. (E) Western blotting indicating the prolonged term hypoxia induces the elevation of p-IκB but without visible change of IκB. (F) Western blotting of the HAF-overexpression induces the accumulation of P65 in the nuclear.

**Figure 3.**
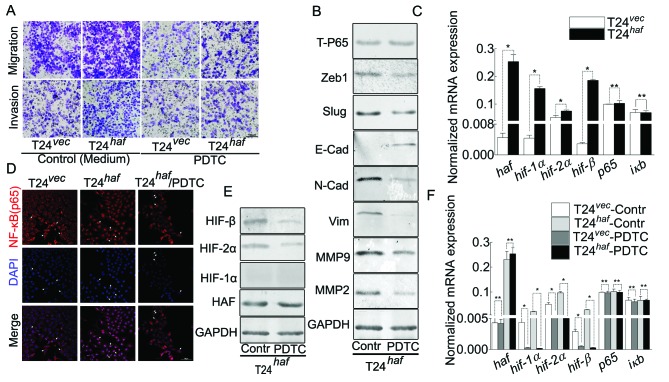
HAF overexpression-induced malignancy, NF-κB nuclear translocation, EMT and alternation of HIFs in T24 cell can be inhibited by PDTC. (A) Representative images of Boyden chamber assay for the attenuated ability of migration/invasion in T24-HAF cells in the presence of PDTC. (B) Western blotting showing the phenomenon induced by PDTC, manifested as the decreased N-cadherin, vimentin, Zeb1, Slug, MMP_2_, MMP_9_ and increased E-cadherin. (C) Real-time PCR indicating that *haf* contributes to the elevation of *hif-1α, hif-2α* and *hif-β* but has no visible effect on *p65* and *iκB*. (D) Immunofluorescence suggesting the nuclear translocation of P65 induced by HAF-overexpression, and its inhibition by PDTC. (E) Western blotting of the elevation of HIF-2α and HIF-β was inhibited by PDTC. (F) Real-time PCR of the HAF-induced elevation of *hif-1α, hif-2α* and *hif-β* was inhibited by PDTC, which still has no visible effect on *p65* and *iκB*.

In order to understand the role of HAF in hypoxia-induced phenotype, the *haf* plasmid was transfected into T24 cells (Fig. 2A), western blotting indicated the extremely increased expression of EMT-related genes and the enhanced ability of migration/invasion (Fig. 3A).

### HAF mimicks the phenomenon occurring to HIFs induced by hypoxia

In order to explore the roles of HAF on HIF-1α, HIFs were detected in the lysate of T24*^haf^* and T24*^vec^* cells. We obtained similar results with the prolonged hypoxic exposure (Fig. 2A). This supplied us evidence that *haf* switched the expression of HIF-1α to HIF-2α in protein, but contributing to the expression of all HIFs in mRNA.

### HAF, consistent with hypoxia, contributes to the degradation of IκB, leading to the activation of NF-κB pathway

As previously reported, in the NF-κB pathway, the degradation of IκB led to the liberation of NF-κB and resulted in the activation of this pathway. As shown in Fig. 2E, hypoxia resulted in decreased IκB and nuclear accumulation of NF-κB (Fig. 1F). In addition, immunofluorescence also indicated the nuclear translocation of NF-κB by hypoxia exposure (Fig. 2B). Based on the phenomenon above, we postulated that HAF may play a role in the degradation of IκB, which was decreased in the T24*^haf^* cells in an unknown way (Fig. 2D). Both western blotting (Fig. 2F) and immunofluorescence (Fig. 3D) suggested that overexpression of HAF in T24 cell induced the nuclear accumulation of NF-κB, leading to the activation of this pathway.

### The role of HAF on EMT and HIFs is inhibited by PDTC or p65 knockdown

Large amount of investigations have pointed out that the NF-κB pathway plays an important role during the process of EMT in the progression of bladder cancer (25). Degradation of IκB led to the activation of this pathway and resulted in the upregulation of EMT-related genes (24,26–30) and HIFs (23). Based on this, we used PDTC, an inhibitor of the NF-κB pathway (31,32), to attenuate the transcription of the target genes of this pathway. In addition, siRNA for knocking down *p65* was used in parallel with PDTC (data not shown). As expected, the nuclear translocation of NF-κB was inhibited (Fig. 3D) and HIFs were all decreased (Fig. 3E), leading to the reversal of EMT induced by HAF-overexpression in T24*^haf^* cells (Fig. 3B), accompanied by the attenuated ability of migration/invasion (Fig. 3A). However, this inhibitor had no significant effect on HAF (Fig. 3E).

### NF-κB contributes to EMT and the enhanced ability of migration/invasion in T24 cells

**Figure 4.**
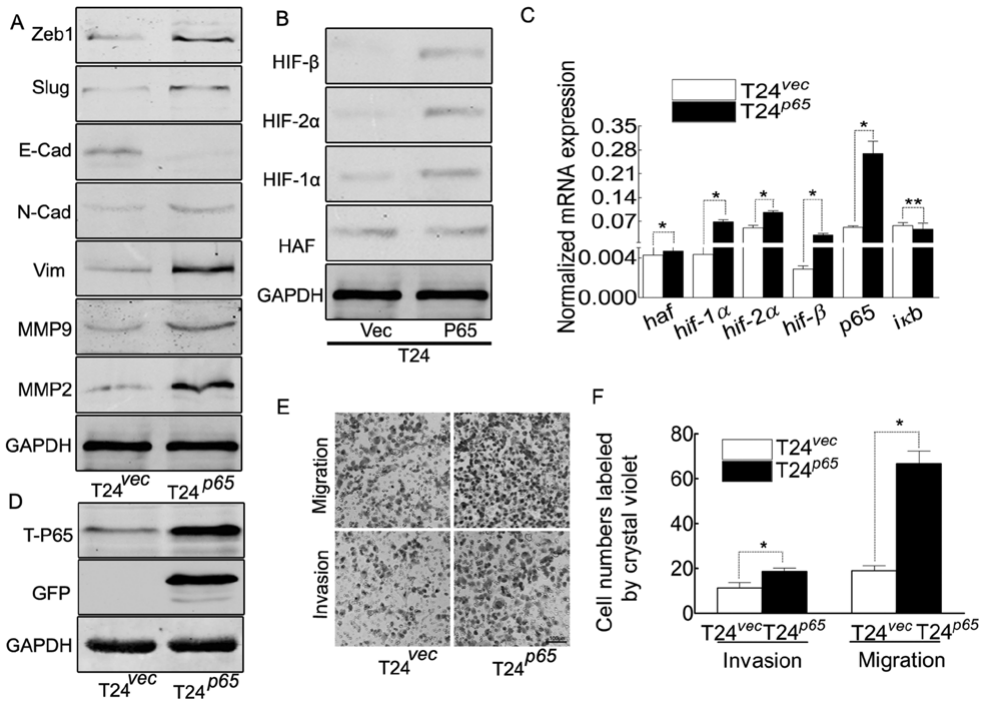
NF-κB-overexpression induces enhanced malignancy and alternation of HIFs in T24 cell. (A) Western blotting showing that p65 gives rise to the elevation of Zeb1, Slug, N-cadherin, vimentin, MMP_2_ and MMP9 but decreased E-cadherin. (B) Western blotting showing that p65 initiates the increase of HIF-1α, HIF-2α and HIF-β, but has no effect on HAF. (C) Real-time PCR showing the elevation of *hif-1α, hif-2α* and *hif-β* induced by *p65*. (D) Western blotting showing the efficiency of P65 transfection and the GFP-fusion protein. (E and F) Boyden chamber assay showing the enhanced ability of migration/invasion induced by P65.

The dimer of *p65/p50* was the critical member among all the combinations of NF-κB family, *p65* was the activator subunit for activating the transcription of the target genes (22,33). In order to explore its roles in T24 cells, the *gfp-p65* was transfected into T24 cells (Fig. 4B–D), resulted in the increasing of HIFs, which gave us evidence that NF-κB contributed to the elevation of HIFs. Thus, at least, NF-κB contributed to EMT and the elevation of HIFs in T24 cells, leading to the enhanced ability of migration/invasion (Fig. 4E and F).

### The switch of HIF-1α to HIF-2α initiates to the different target genes, accompanied by the enhanced ability of migration/invasion in T24 cells

**Figure 5.**
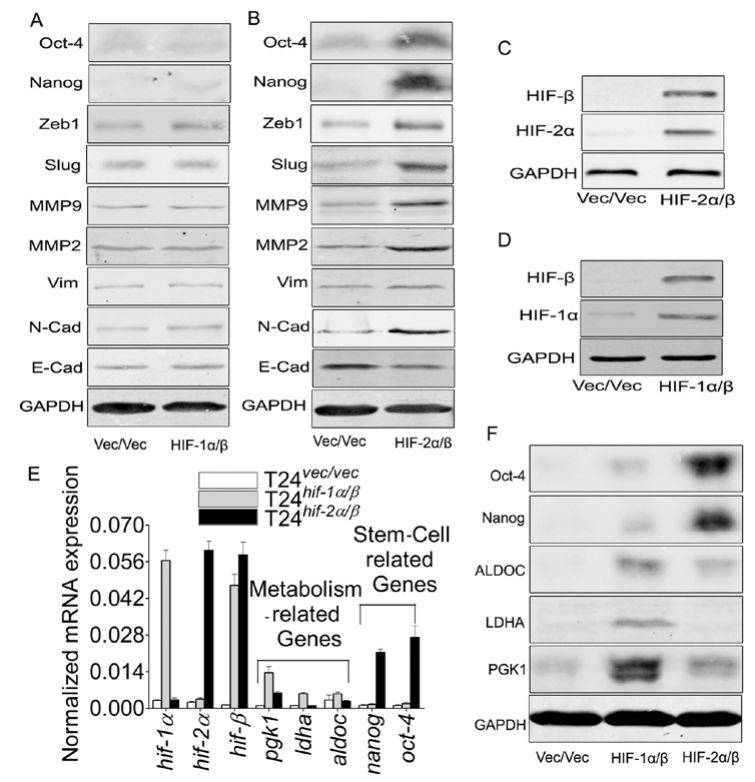
Dimer of *hif-1α/β* and *hif-2α/β* has different target gene profiles in T24 cell. (A) Western blotting indicating no visible difference of EMT related genes plus stem cell markers induced by HIF-1α/β and Vec/Vec. (B) Western blotting indicating the elevation of Nanog, Oct-4 and EMT-related genes in the T24HIF-2α/β compared with the Vec/Vec. (C and D) Western blotting showing the efficiency of cotransfection of HIF-1α/β or HIF-2α/β. (E) Real-time PCR showing the different target genes in the three subclones. *Hif-1α, hif-2α* and *hif-β* are all elevated in T24*^hif-1α/β^* and T24*^hif-2α/β^*. Specifically, *hif-1α/β* results in the induction of *pgk1, ldha* and *aldoc, hif-2α/β* targets *nanog* and *oct-4*. (F) Western blotting showing the discrepancy of targets induced by the two dimers. Clearly, HIF-1α/β affect the metabolism-related genes, but the HIF-2α/β emphasizes the stem-cell related genes.

**Figure 6.**
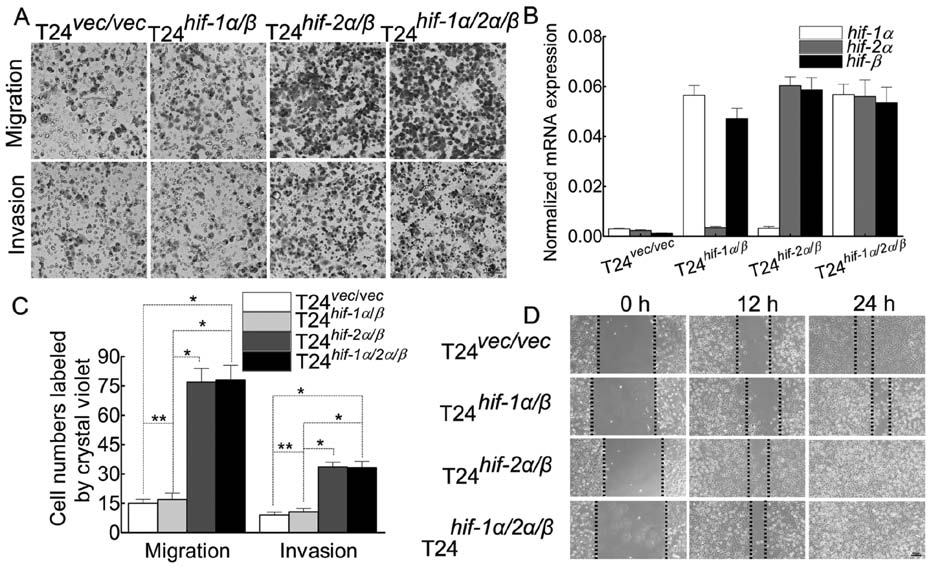
The migration/invasive ability *in vitro*. (A and C) Boyden chamber assay indicating the enhanced ability of migration/invasion in T24*^hif-2α/β^*. (B) Real-time PCR showing the efficiency of cotransfection of *hif-1α/2α/β* in the T24 cell. (D) Would healing assay indicating the enhanced ability of malignancy in T24*^hif-2α/β^*.

As has been reported (6), although HIF-1α and HIF-2α share several common targets, both isoforms may induce distinct transcriptional target genes (16). In order to observe this discrepancy in T24 cells, by cotransfection of the two subunits into the T24 cells (Fig. 5C and D), we proved the discrepancy of target genes (Fig. 5A, B, E and F) and the ability of migration/invasion (Fig. 6A, C and D). Herein we chose the cotransfection of the β subunit with α subunit because α subunit must couple with β subunit, which is greatly lower in T24 cells (Fig. 1D), to activate their target genes.

## Discussion

Hypoxia, a characteristic feature of solid tumors, has emerged as a pivotal factor of the tumor since it can activate the related genes of the tumor cells in order to adapt to the microenvironment to promote tumor progression and resistance to therapy (34). Among large numbers of genes, the key candidate is widely accepted to be the HIF family, which can be regulated by an oxygen- or pVHL-dependent mechanism. Recently, HAF is reported to regulate the HIFs both in an oxygen- and pVHL-independent way. In the present study, we provide evidence that HAF contributes to the degradation of HIF-1α directly and promotes the transcriptional activation of all HIFs by activating the NF-κB pathway, leading to the elevation of HIFs in mRNA indirectly. The combination of two aspects results in the switch of HIF-1α/β to HIF-2α/β followed by the activation of different target genes, leading to the malignant behavior of tumor cells. To our knowledge, this is the first study involved in the NF-κB pathway during the process of HAF induced switch of HIF-1α/β to HIF-2α/β in bladder cancer cells.

The HIF-α/β heterodimer binds to the hypoxic response elements (HREs) of target genes on hypoxia (35) to promote their expression, leading to the tumor behavior change to adapt to the hypoxic environment. We noted that on the different time-point of hypoxia, the expressions of the HIFs are different (Fig. 1D and E). It is reported that HIF-1α but not HIF-2α is more sensitive to the oxygen, and can be degradated within 5 min (8,21) in normal atmosphere, but this protein can keep the stable status in hypoxia. However, compared to the reported results (4,36,37), our data indicate that this stable status on hypoxia can be destroyed in protein, but not mRNA by the prolonging time of hypoxic exposure. Compared with HIF-1α, hypoxia induces the elevation of HIF-2α and HIF-β instead of reduction.

Hypoxia induces the expression of HAF, accompanied by the different changes of HIFs in T24 and J82 cells (J82 cell data not shown), the process of which could be mimicked by HAF overexpression. Our results are consistent with the investigation (16) that HAF contributes to the switch of HIF-1α/β to HIF-2α/β. The importance of this switch is the different target genes of the two dimers. It is reported that HIF-1α/β preferentially induces glycolytic enzyme genes (8,38,39), but HIF-2α/β induces genes involved in invasion and are stem cell related (8,16). Our data in T24 cells provide evidence on this point that HIF-1α/β promotes the expression of metabolism-related genes (Fig. 5E and F), but HIF-2α/β affect the induction of EMT and migration/invasion-related genes, also including the stem cell-related genes (Fig. 5B, E and F). This discrepancy indicates the initiation of the differing ability of migration/invasion (Fig. 6A, C and D). In order to explore whether HIF-1α affects the HIF-2α/β induced malignant behavior of T24 cells, we transfect *hif-1α* into T24*^hif-2α/β^* cells (Fig. 6). To our surprise, there is almost no discrepancy of malignancy between T24*^hif-2α/β^* and T24*^hif-1α/2α/β^* cells, which indicates that there must be a more complex mechanism during this process. Besides the above, the enhanced ability of migration/invasion can be induced also by the NF-κB pathway directly.

In bladder cancer, Levidou *et al* (40) demonstrated that NF-κB nuclear expression emerged as an independent prognosticator of adverse significance. While, this nuclear expression was regarded also as a marker of activation of NF-κB pathway, in the process where the degradation of IκB is the key step (30). We noted that either in hypoxia or in the T24*^haf^* cells, IκB is decreased, accompanied by the nuclear translocation of NF-κB (Figs. 1E and F, 2B, D, E and F and 3D), which means, at least, HAF is one of the factors contributing to the degradation of IκB in an unknown mechanism and leads to the activation of the NF-κB pathway.

It was reported (39,41) that HIF-1α is a target gene for NF-κB, and the promoter of HIF-1α contained a NF-κB binding site (42,43). Furthermore, NF-κB also controlled HIF-β directly and HIF-2α indirectly, making NF-κB a key regulator of the HIF family (1,23,44). Our activation or inhibition results also illustrate this point. The activation or inhibition of the NF-κB pathway is significantly accompanied by the inverse EMT related markers and the ability of migration/invasion in our T24 cells.

**Figure 7.**
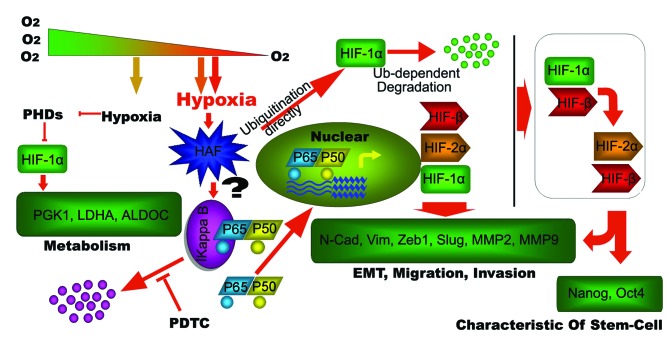
Schematic diagram as summary of the present investigation. Short-term hypoxia leads to the stabilization of HIF-1α, targeting the metabolism-related genes, such as PGK1, ALDOC and LDHA, to adapt to the hypoxic environment. The prolonged hypoxia is more sophisticated. Firstly, this hypoxia induces HAF. On the one hand, the induced HAF contributes to the degradation of IκB in an unknown way, leading to the activation of NF-κB pathway, which can be inhibited by PDTC resulting in the increase of target genes, such as HIF-1α, HIF-2α and HIF-β. On the other hand, HAF leads to the degradation of HIF-1α directly. The combination of the two aspects result in the switch of HIF-1α to HIF-2α, leading to the activation of migration/invasion and stem-cell related genes to maintain invasive and self-renewal capacity of tumor cells.

In conclusion, our present study summary is shows in Fig. 7. There are still several problems to be explored in our following work. For instance, HAF is reported as an E3 ubiquitin ligase that binds and ubiquitinates HIF-1α by an oxygen and pVHL-independent mechanism, but our results indicate that this protein also plays roles in the phosphorylation of IκB (Fig. 2D). Phosphorylation of IκB, which also occurs in hypoxia (Fig. 2E), is the first and one of the prerequisite steps for its degradation. It seems that HAF can induce the phosphorylation followed by ubiquitination of IκB in some way, resulting in the activation of the NF-κB pathway in T24 cells. In addition, we note that total P65 was elevated either by hypoxia exposure (Fig. 1A) or by HAF-overexpression (Fig. 2A), but this elevation seems not to be inhibited by PDTC (Fig. 3B), while, the N-P65 can be inhibited by PDTC. This gives us a clue there must be some sophisticated mechanisms involved in this process. We found that HAF and NF-κB pathway play key roles in the switch of HIF-1α/β to HIF-2α/β in hypoxia, thus providing a blueprint for future investigation of this signal pathway.

## Figures and Tables

**Table I.** The antibodies used.

Gene ID	Antibody	Dilutions	Species	Supplied by
NM_004360.3	E-cadherin	1:600	Homo	Santa Cruz
NM_001792.3	N-cadherin	1:300	Homo	Santa Cruz
NM_003380.3	Vimentin	1:300	Homo	Santa Cruz
NM_003068.4	Slug/Snail2	1:400	Homo	Santa Cruz
NM_001174096.1	Zeb1	1:300	Homo	Santa Cruz
NM_001530.3	HIF-1α	1:500	Homo	Santa Cruz
NM_001430.4	HIF-2α	1:300	Homo	Santa Cruz
NM_001668.3	HIF-β	1:300	Homo	Santa Cruz
NM_005146.4	HAF	1:400	Homo	Santa Cruz
NM_001145138.1	P65	1:300	Homo	Santa Cruz
NM_004530.4	MMP2	1:400	Homo	Santa Cruz
NM_004994.2	MMP9	1:400	Homo	Santa Cruz
NM_002046.4	GAPDH	1:15,000	Homo	Santa Cruz
NM_024865.2	Nanog	1:400	Homo	Millipore
NM_001173531.1	Oct-4	1:300	Homo	Millipore
NM_020529.2	IκB	1:400	Homo	Santa Cruz
NM_020529.2	p-IκB	1:300	Homo	Santa Cruz
	GFP	1:300		Santa Cruz
NM_005325.3	Hist1H1a	1:300	Homo	Santa Cruz

**Table II.** Primers for real-time PCR and siRNA sequence.

Gene ID	Gene		Primers
NM_001145138.1	p65	Forward	ACGAATGACAGAGGCGTGTATAAGG
		Reverse	CAGAGCTGCTTGGCGGATTAG
NM_001197325.1	HIF-β	Forward	CTCTGTGGACCCAGTTTCTGTGA
		Reverse	CAGGCCTTGATGTAGCCTGTG
NM_001530.3	HIF-1α	Forward	TTGCTCATCAGTTGCCACTTCC
		Reverse	AGCAATTCATCTGTGCTTTCATGTC
NM_001430.4	HIF-2α	Forward	CATGCGCTAGACTCCGAGAACA
		Reverse	GCTTTGCGAGCATCCGGTA
NM_005146.4	HAF	Forward	AAGTACAGCCGGAGGGAGGAATAC
		Reverse	TTCATCTTGCCTGAGCCCTTG
NM_002046.4	GAPDH	Forward	AACAGCGACACCCATCCTC
		Reverse	CATACCAGGAAATGAGCTTGACAA
NM_020529.2	IκBα	Forward	GATCCGCCAGGTGAAGGG
		Reverse	GCAATTTCTGGCTGGTTGG
NM_024865.2	Nanog	Forward	CTAAGAGGTGGCAGAAAAACA-3
		Reverse	CTGGTGGTAGGAAGAGTAAAGG
NM_001173531.1	Oct-4	Forward	TTGGGCTAGAGAAGGATGTGGTT
		Reverse	GGAAAAGGGACTGAGTAGAGTGTGG
NM_005165.2	ALDOC	Forward	CGTCCGAACCATCCAGGAT
		Reverse	CACCACACCCTTGTCAACCTT
NM_000291.3	PGK1	Forward	CTGTGGTACTGAGAGCAGCAAGA
		Reverse	CAGGACCATTCCAAACAATCTG
NM_001135239.1	LDHA	Forward	TGCCTACGAGGTGATCAAGCT
		Reverse	ATGCACCCGCCTAAGGTTCTT
NM_001145138.1	p65-siRNA	Antisense	AAGAGCATCATGAAGAAGAGTCCTGTCTC
		Sense	AAACTCTTCTTCATGATGCTCCCTGTCTC
